# An entropy-based study of Simplification in ChatGPT translations compared to neural machine translation and human translation across genres

**DOI:** 10.1371/journal.pone.0339762

**Published:** 2025-12-31

**Authors:** Guangyuan Yao, Lingxi Fan

**Affiliations:** 1 School of Foreign Languages, Central South University, Changsha, Hunan, China; 2 Hangzhou International Innovation Institute, Beihang University, Hangzhou, Zhejiang, China; National University of Malaysia Faculty of Education: Universiti Kebangsaan Malaysia Fakulti Pendidikan, MALAYSIA

## Abstract

This study investigates the phenomenon of simplification in Chinese-to-English translation across Human Translation (HT), neural machine translation (NMT), and large language model (LLM)-based translation, ChatGPT as an example. Employing entropy-based metrics (unigram entropy and Part-of-Speech (POS) entropy) to assess lexical and syntactic complexity, the research analyzes translations across three genres: political texts, fiction, and academic. Findings reveal that political and academic texts exhibit lexical simplification, and texts of all genres show a syntactic simplification trend, with the simplified degree varying across translation modes. While genre exerts minimal influence on lexical complexity, it significantly impacts syntactic complexity, with academic texts showing the lowest and fiction the highest complexity levels. Notably, ChatGPT’s translations consistently exhibit greater lexical complexity, as evidenced by higher unigram entropy scores compared to those of Neural Machine Translation. These results challenge the notion of simplification as a universal feature of translation, instead highlighting its probabilistic nature influenced by translation mode and genre. The study underscores the efficacy of entropy-based measures in capturing nuanced differences in translation complexity and advocates for a modal approach to translation studies that accounts for the unique characteristics of various translation methods.

## 1. Introduction

The rapid proliferation of machine translation (MT) technology, evolving from statistical methods to sophisticated neural networks (NMT) [1] and now incorporating large language models (LLMs) like ChatGPT, is profoundly impacting translation practices and research paradigms [2–4]. While MT systems are increasingly integrated into diverse communication workflows, a thorough understanding of the linguistic characteristics of their output, particularly in comparison to Human Translation (HT), is still developing.

A cornerstone of corpus-based translation studies (CBTS) has been the quest for “translation universals” (TUs). First systematically conceptualized by Baker [5], TUs are defined as linguistic features “which typically occur in translated text rather than original utterances and which are not the result of interference from specific linguistic systems” (p. 243). Crucially, Baker maintained that these universals are the outcome of constraints and conditions intrinsic to the translation process itself, such as the pressure to disambiguate meaning or manage cognitive load, rather than being specific to any language pair [5, p. 246]. Furthermore, this foundational hypothesis assumes that such features are largely uniform across different cultures and translation contexts. This notion aligns with earlier concepts like “the third language” [6] and “the third code” [7], suggesting that translated texts possess unique characteristics distinct from both source and target languages. Key hypothesized TUs include explicitation [8,9], simplification [10,11], normalization or conservatism [12–14], and source language shining-through [15]. Among these, the simplification hypothesis has been extensively, yet controversially, investigated [16]. It proposes that translators, consciously or unconsciously, tend to produce texts that are linguistically simpler than non-translated target language texts [12, p. 176; 10, p. 119]. The foundational empirical investigation of this hypothesis was conducted by Laviosa [11,17], who operationalized simplification across two primary dimensions. To test for lexical simplification—a reduction in vocabulary richness and specificity—she employed a suite of metrics including lexical density, type-token ratio (TTR), list head, and core vocabulary coverage. To assess syntactic simplification—a move towards less complex or varied sentence structures—she primarily used mean sentence length. However, subsequent research across various language pairs, genres, modalities, and directions has yielded conflicting results. While lexical simplification is frequently observed [18,19], findings for syntactic simplification are inconsistent [11,20,21]. This suggests that simplification is a complex phenomenon influenced by multiple factors, including the specific linguistic features measured [22].

Research into simplification in MT output is comparatively nascent but equally complex. Lapshinova-Koltunski [23] found mixed evidence in early MT systems compared to HT. Studies comparing NMT and HT have also shown divergent patterns; for example, Niu and Jiang [24] found NMT (Google, DeepL) to be more lexically simplified than HT across several genres, while Luo and Li [25] found that WeChat Translate exhibited greater lexical range and variety than expected under the simplification hypothesis. Furthermore, while some studies suggest MT shares simplification traits with HT [26], others highlight distinct characteristics, such as potentially stronger source language shining-through in MT [27,28].

Two significant gaps persist in simplification research. Firstly, the influence of genre, though acknowledged as important [21,29,30], is often under-investigated, limiting the generalizability of findings. Secondly, while MT has been compared separately to HT or original texts, comparisons involving all three, especially including the latest LLM-based systems like ChatGPT, are scarce. Understanding how these new systems perform relative to both human benchmarks and established NMT systems regarding TUs is essential.

To address these gaps and move beyond the limitations of traditional, often conflicting, simplification metrics, this study adopts an entropy-based approach. Rooted in Shannon’s [31,32] information theory, entropy provides a global measure of the complexity, diversity, and predictability of linguistic units within a text. It offers advantages over isolated indicators by being less sensitive to text length and capturing distributional patterns [33–35]. Following Liu et al. [36], we utilize unigram entropy (based on word forms) to assess lexical complexity and Part-of-Speech (POS) entropy to gauge structural or syntactic complexity. In this study, simplification is conceptualized as the reduction of lexical and/or syntactic complexity in translated texts compared to non-translated target language texts. Lexical simplification is defined as a reduction in vocabulary diversity and complexity, often measured through metrics such as unigram entropy. Syntactic simplification refers to the reduction in sentence complexity, which can include factors such as sentence length, subordination, and syntactic structures. Both forms of simplification are measured using entropy-based metrics: unigram entropy to capture lexical complexity and Part-of-Speech (POS) entropy to assess syntactic complexity.

This study systematically investigates lexical and syntactic simplification (operationalized via entropy) in English translations of Chinese texts generated by ChatGPT, comparing them with outputs from established NMT systems (Google Translate) and professional human translators across three genres: fiction, political texts, and academic. Non-translated English originals serve as the baseline reference. Specifically, we aim to answer:

1. Do ChatGPT translations, NMT, and HT exhibit simplification (lower lexical and/or syntactic entropy) compared to non-translated original texts across different genres?
2. How do the simplification patterns (lexical and syntactic entropy) compare among ChatGPT, NMT, and HT?
3. Does genre significantly influence the entropy patterns observed across the different text varieties (ChatGPT, NMT, HT, Originals)?

By employing a robust entropy-based methodology within a multi-genre, multi-variety comparable corpus framework, this research seeks to provide nuanced insights into the simplification universal in the context of contemporary MT, particularly LLMs. The findings aim to contribute to the refinement of TU theory, enhance our understanding of machine translationese, and offer potential implications for MT development and evaluation.

## 2. Literature review

The field of corpus-based translation studies (CBTS) has significantly advanced our understanding of the nature of translated language, largely driven by the pursuit of “translation universals” (TUs). Baker [5] initially proposed TUs as linguistic features characteristic of translated texts, which differentiate them from original utterances. She maintained that they arise from the translation process itself, rather than from direct linguistic interference. This concept aligns with notions like “the third language” [6] and “the third code” [7], suggesting translation fosters a unique linguistic system. Key hypothesized TUs include explicitation, normalization, source language shining-through, and the central focus of this paper: simplification [8,12–15,17]. The simplification hypothesis posits that translators tend, consciously or unconsciously, to make the target text linguistically simpler than comparable non-translated texts, possibly to enhance clarity or reduce cognitive load [10,12]. This simplification can manifest at lexical, syntactic, and stylistic levels.

Much of the empirical work on simplification stems from Laviosa’s [11,17] comparative analyses using the English Comparable Corpus (ECC). She operationalized simplification through several metrics: lexical density (content words vs. function words), type-token ratio (TTR, measuring lexical variety), core vocabulary coverage and list head coverage (measuring reliance on frequent words), and mean sentence length (MLS). Her studies found compelling evidence for lexical simplification in translated English narrative prose (lower density, higher frequency word use) compared to originals. However, syntactic simplification, proxied by MLS, showed genre dependency: translated political texts featured shorter sentences (simplification), while translated fiction featured longer sentences (complexification).

Subsequent research has painted a more complex picture, confirming the contingency of simplification. Lexical simplification metrics often, but not universally, support the hypothesis. For instance, studies involving English and typologically distant languages like Chinese have shown lower lexical density [20] but sometimes similar lexical variety (TTR) compared to native originals [37]. Cvrček and Chlumská [18] found reduced lexical diversity (TTR) in translated Czech literary texts. However, directionality matters significantly; Williams [38] observed simplification in English translated from French but complexification in French translated from English using the same metrics. Ferraresi et al. [22], studying English translated from Italian in parliamentary proceedings, also found evidence contradicting lexical simplification, noting higher lexical density.

Evidence for syntactic simplification is even more inconsistent. While Laviosa [11] found shorter sentences in translated news, her findings for fiction [17] and studies like Xiao [20] and Xiao and Yue [37] on Chinese translations showed longer sentences or no significant difference. Liu and Afzaal [16], however, employed a wider range of 14 syntactic complexity indices (beyond just MLS) and found that overall, translated English (from Chinese) was indeed syntactically simpler than native English across multiple measures related to subordination and sentence structure, although genre variations were present (e.g., translated fiction was more complex). This suggests that reliance solely on MLS might obscure more nuanced syntactic patterns.

Furthermore, genre consistently emerges as a critical variable. Kruger and van Rooy [29] found simplification tendencies varied between informational and creative genres in translated South African English. Liu and Afzaal [16] and Niu and Jiang [24] also confirmed that genre significantly impacts both lexical and syntactic complexity patterns in translated texts. This accumulating evidence indicates that simplification in HT is not an absolute universal but a tendency influenced by language pair, directionality, genre, register, and potentially the specific linguistic level being examined.

Investigating simplification in MT introduces further complexities, as algorithmic processes replace human cognitive ones. The applicability of hypotheses rooted in Human Translation behaviour (like minimizing effort) is questionable. Early research comparing MT (often SMT) with HT yielded mixed results. Lapshinova-Koltunski [23], in her English-to-German study, found that both MT and HT exhibited simplification via lower standardized TTR compared to originals. However, MT showed higher lexical density than HT, leading her to conclude that simplification wasn’t fully confirmed for MT, while normalization seemed a more defining characteristic.

Studies focusing on NMT continue this trend of mixed findings. Lexical simplification results vary: Han and Jiang [39] found Baidu Translate (NMT) had higher TTR but lower lexical density than HT. Luo and Li [25], examining WeChat Translate (Chinese-English NMT), found higher STTR and lexical density compared to native English, contradicting the simplification hypothesis for their data. Conversely, Niu and Jiang [24], using TTR variants less sensitive to text length (vocd and MTLD), found Google Translate and DeepL produced lower lexical diversity (stronger simplification) than both HT and English originals across novels, government documents, and academic abstracts. They also noted MT was even more lexically simplified than HT. Research on syntactic simplification in MT is less common but growing. Bizzoni et al. [26] suggested SMT tended towards syntactic simplification, while NMT outputs were closer to HT in complexity. Studies specifically measuring syntactic complexity indices are needed to confirm these observations for current NMT and emerging LLM systems. The divergent results suggest that MT simplification patterns might differ significantly from HT and vary based on the MT paradigm (SMT vs. NMT vs. LLM), the specific system, the metrics used, and, as highlighted by Niu and Jiang [24] and Wang and Jiang [40], the genre of the text being translated.

The inconsistencies observed using traditional metrics (like TTR’s sensitivity to text length or MLS’s genre variability) highlight the need for more robust, global measures of linguistic complexity that can capture simplification across different levels. Shannon’s information entropy [31,32] offers such a framework. By measuring the average predictability of linguistic units, entropy provides an information-theoretic quantification of diversity and complexity; lower entropy signifies higher predictability and thus simplification.

Liu et al. [36] successfully applied this methodology to study simplification in HT (translated Chinese vs. native Chinese). They used unigram entropy (based on word forms) as an indicator of lexical complexity and POS entropy (based on Part-of-Speech tag distribution) as an indicator of syntactic/structural complexity. Their crucial finding was a dissociation: translated Chinese exhibited lexical simplification (lower unigram entropy) but syntactic complexification (higher POS entropy) compared to originals. This demonstrates entropy’s potential to reveal nuanced, multi-level linguistic profiles that simpler metrics might miss. This entropy-based approach has also been effective in differentiating translated from non-translated texts using machine learning [41] and analysing complexity across genres [42].

Despite the growing body of research, significant gaps remain, particularly concerning the latest generation of MT systems based on LLMs like ChatGPT. It is unknown whether these systems, with their advanced architectures and vast training data, exhibit simplification patterns similar to NMT, HT, or originals. Furthermore, the interaction between translation modes (LLM vs. NMT vs. HT) and genre in shaping simplification requires systematic investigation. Finally, the conflicting results from previous studies using traditional metrics necessitate the application of more robust measures like entropy.

This study addresses these gaps by employing unigram entropy to systematically compare the lexical simplification profiles of ChatGPT translations, established NMT outputs (Google Translate), Human Translations, and non-translated English originals. By analysing these text varieties across three distinct genres, we aim to provide the first entropy-based assessment of simplification in LLM translations relative to other modalities, while rigorously investigating the moderating influence of genre.

## 3. Methodology and data

### 3.1. Corpora

This study adopts a comparative corpus-based approach to examine simplification patterns across different translation modes and genres, following the methodology established by Laviosa [17] and refined by Kruger and van Rooy [29]. As is demonstrated in Table 1, the corpus consists of texts from three genres (political texts, novel, and technology) in four variants: original English texts, Human Translations from Chinese to English, Neural Machine translations, and ChatGPT translations. This multi-genre design aligns with recent research demonstrating the importance of genre variation in translation studies [16,21]. Text selection followed the criteria outlined by Bernardini et al. [19] to ensure comparable representation across variants. Human Translations were produced by professional translators with over five years of experience in Chinese-English translation, adhering to quality standards described by Delaere et al. [30]. Google translations were generated using Neural Machine Translation System, while ChatGPT translations were generated using ChatGPT-4, following protocols established in recent machine translation research [25,43]. The prompt was ‘Translate into English.’ Translations were obtained via the official website under default settings, with no manual selection among alternatives and no multiple-generation attempts.

**Table 1:** Details of the corpus constructed.

Genre	Text Variety		Composition	Text Count	Overall Size	Mean Size
Political texts	Original English		*the State of the Union Address*	50	112,364	2247
	Translations	Human Translator	Report on the *Work of the Government* and the translations	50	119464	2389
		Google Translate		50	112006	2240
		GPT		50	96817	1936
Fiction	Original English		*Then We Came to the End* by Joshua Ferris; *Let the Great World Spin* by Colum McCann; *The Sense of an Ending* by Julian Barnes	50	115750	2315
	Translations	Human Translator	*Red Sorghum* by Mo Yan, translated by Howard Goldblat; *Decoded* by Mai Jia, translated by Olivia Milburn and Christopher Payne; *Faded Dreams* by Jia Pingwa, translated by Howard Goldblatt	50	135308	2706
		Google Translate		50	132325	2647
		GPT		50	125857	2517
Academic	Original English		Abstracts from the *Target* and *Perspective* journals	50	102483	2050
	Translations	Human Translator	Abstracts from *Chinese Science and Technology Translators Journal (CSTTJ)*	50	88828	1777
		Google Translate		50	102868	2057
		GPT		50	87,861	1757

### 3.2. Entropy measures and statistical analysis

Building on Shannon’s [31,32] information theory framework and its successful application in translation studies [36], two types of entropy measures were employed to analyze linguistic complexity. The selection of entropy-based measures is supported by their proven effectiveness in capturing both lexical and structural complexity [44,45].

The first measure, unigram entropy, is calculated using Formula (1), where p(x) represents the probability of each word (unigram) occurring in the text. We foreground this measure to capture lexical complexity for several reasons, particularly in relation to traditional metrics.

Unigram entropy is most directly a measure of lexical diversity. As demonstrated in previous research, higher entropy values indicate greater lexical richness and variation [33,34]. However, unlike traditional metrics such as Type-Token Ratio (TTR), entropy is less sensitive to text length and, crucially, accounts for the evenness of the vocabulary’s distribution. A text where many different words are used with similar frequency will yield a higher entropy score than one dominated by a few recurring words, even if their raw word counts are similar.

While entropy does not directly measure lexical sophistication (i.e., the use of rare or advanced words), it often serves as a powerful proxy. A text employing a wider range of sophisticated vocabulary will naturally have a more uniform probability distribution among its words, leading to higher entropy. Finally, it is important to distinguish entropy from lexical density, which is concerned with the ratio of content-to-function words. A text can have high lexical density but low entropy if the same few nouns and verbs are used repeatedly. Therefore, by adopting unigram entropy, we employ a single, robust metric that encapsulates key aspects of lexical diversity and correlates with sophistication, thus providing a more holistic and distributionally sensitive assessment of lexical complexity than isolated traditional measures. The second measure, POS entropy, follows the Formula (2) as follows, where p(pos) represents the probability of each part-of-speech tag occurring in the text. This measure reflects syntactic complexity, with higher values indicating more diverse grammatical structures, a method validated by recent studies in translation complexity [36,46]. To eliminate the influence of text length, we used the normalized entropy measure [47].


H = −Σ p(x) log p(x) (1)

H = −Σ p(pos) log p(pos) (2)

Following established practices in corpus-based translation studies [16,19], statistical analysis began with normality testing using Shapiro-Wilk test and homogeneity of variance testing using Levene’s test. Statistical analysis began with normality testing using the Shapiro-Wilk test for the Normalized Unigram Entropy and Normalized POS Entropy. For Normalized Unigram Entropy, the test produced a W value of 0.987 and a p-value of < 0.001, indicating that the data does not follow a normal distribution (p < 0.05). Similarly, for Normalized POS Entropy, the Shapiro-Wilk statistic was 0.965 and the p-value was <  0.001, which also rejects the assumption of normality (p < 0.05). Furthermore, Levene’s test for homogeneity of variance was conducted. For Normalized Unigram Entropy, the W statistic was 0.318 with a p-value of 0.58, indicating that the variance is homogeneous (p > 0.05). Similarly, for Normalized POS Entropy, the W statistic was 1.173 with a p-value of 0.32, also supporting homogeneity of variance (p > 0.05). The Kruskal-Wallis test is particularly suitable for situations where the data do not follow a normal distribution, as it does not require the assumption of normality. Moreover, the Kruskal-Wallis test is designed to compare three or more independent groups, making it appropriate for evaluating the differences in Normalized Unigram Entropy and Normalized POS Entropy across the various translation methods (ChatGPT, Human Translation, and Google Translate). This test operates by ranking the data from all groups and analyzing the differences in ranks, rather than the raw data values, which further mitigates the effects of non-normality. As such, it offers a reliable method for comparing the distributions of entropy values between translation modes when the normality assumption is violated.

Text preprocessing and analysis followed protocols established in recent translation studies [23,48]. Texts were processed using standard NLP tools for tokenization and POS tagging, implementing methods validated by previous research [36]. Entropy calculations were implemented in Python, following computational approaches established by Takahira et al. [34], and statistical analyses were performed using R statistical software, adhering to practices recommended by De Sutter and Lefer [49]. This comprehensive methodological approach enables systematic comparison of simplification patterns across different translation modes and genres while maintaining statistical rigor.

## 4. Findings

The data presented in the Table 2 shows the mean normalized unigram entropy and mean normalized POS (Part-of-Speech) entropy across different corpora (Original, Human, Google, ChatGPT) and genres (Political Texts, Fiction, Academic). Normalized unigram entropy measures the lexical diversity and complexity of a text. Higher values indicate a greater diversity of vocabulary used, while lower values reflect more repetition or a less varied vocabulary.

**Table 2:** Mean normalized unigram entropy and POS entropy of this corpus.

Corpus	Genre	Unigram Mean	Std. Deviation	POS Mean	Std. Deviation
Original	Political Texts	0.8674	0.0125	0.8247	0.0149
	Fiction	0.8654	0.0101	0.8093	0.0176
	Academic	0.8559	0.0099	0.7523	0.0152
	Total	0.8629	0.0122	0.7954	0.0355
Human	Political Texts	0.8383	0.0116	0.7788	0.0191
	Fiction	0.8540	0.0093	0.8052	0.0175
	Academic	0.8499	0.0084	0.7288	0.0142
	Total	0.8474	0.0114	0.7709	0.0335
Google	Political Texts	0.8376	0.0107	0.7583	0.0189
	Fiction	0.8481	0.0084	0.7940	0.0248
	Academic	0.8322	0.0059	0.7214	0.0132
	Total	0.8393	0.0099	0.7579	0.0337
ChatGPT	Political Texts	0.8665	0.0142	0.7645	0.0185
	Fiction	0.8576	0.0089	0.7963	0.0232
	Academic	0.8669	0.0084	0.7391	0.0134
	Total	0.8637	0.0119	0.7666	0.0259

To statistically assess the differences in linguistic complexity, a Kruskal-Wallis H test was conducted to determine if significant differences existed for Normalized Unigram Entropy and Normalized POS Entropy across the four corpora (Original, Human, Google, ChatGPT) and the three genres (Political Texts, Fiction, Academic). The significance level was set at α = .05. For all significant results, a Dunn’s post-hoc test with Bonferroni correction was used for pairwise comparisons.

Starting with Normalized Unigram Entropy, which measures lexical diversity, the Original (M = 0.8629) and ChatGPT (M = 0.8637) corpora demonstrate the highest and nearly identical levels of lexical complexity, indicating the most diverse vocabulary. In contrast, Human (M = 0.8474) and particularly Google (M = 0.8393) translations show a noticeable reduction in this metric, suggesting a degree of lexical simplification compared to the original texts. Among the genres within the Original texts, Political texts (M = 0.8674) exhibits the highest lexical diversity. However, this pattern shifts in translated texts, where Fiction tends to show higher diversity in Human (M = 0.8540) and Google (M = 0.8481) translations. The lowest lexical diversity is observed in Google’s translations of Academic (M = 0.8322), pointing to significant vocabulary simplification in that context.

Moving on to Normalized POS Entropy, which reflects syntactic complexity, the Original corpus again shows the highest POS entropy (M = 0.7954), indicating the most complex sentence structures. A clear trend of syntactic simplification is visible across the other corpora, with Human translations (M = 0.7709) being simpler than the original, followed by ChatGPT (M = 0.7666), and Google translations (M = 0.7579) exhibiting the least syntactic complexity. A consistent pattern emerges across genres: in the Original texts, Political texts (M = 0.8247) has the most diverse syntax. However, in all other corpora—Human, Google, and ChatGPT—Fiction texts consistently display the highest syntactic complexity. Conversely, Academic consistently shows the lowest POS entropy across all four corpora, suggesting that this genre, regardless of origin or translation method, employs simpler sentence constructions compared to Political texts and Fiction in this dataset. This simplification is most pronounced in Google’s Academic translations (M = 0.7214).

**Fig 1:**
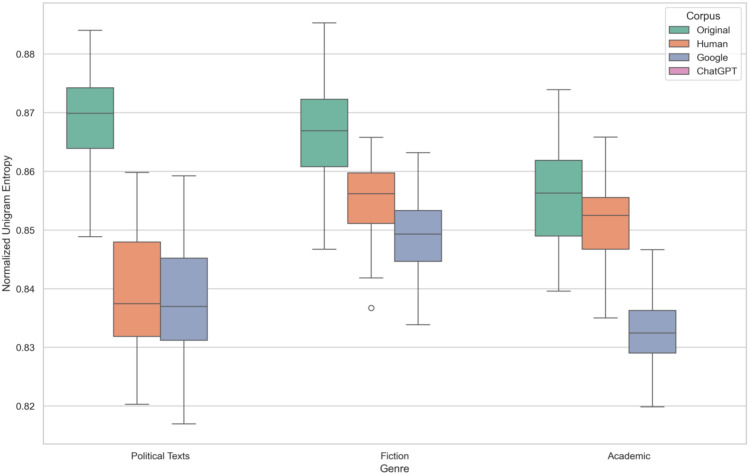
Unigram Entropy Across Corpus and Genres.

As illustrated in Fig 1, the distribution of Normalized Unigram Entropy varies distinctly across both corpora and genres. This visualization corroborates the statistical findings, highlighting several key trends in lexical diversity.

First, the Original and ChatGPT corpora consistently exhibit the highest median entropy values across all genres, with their interquartile ranges largely overlapping, visually supporting the non-significant difference found in the post-hoc analysis. In contrast, Human and, more notably, Google translations show a clear downward shift in entropy, indicating lexical simplification. The Google corpus consistently occupies the lowest position in every genre, suggesting it produces the least lexically diverse texts.

Second, when examining the effect of genre, Political texts generally display the highest lexical diversity, particularly within the Original and ChatGPT corpora. The median entropy for Fiction and Academic is comparatively lower, aligning with the statistical result that Political texts are significantly more diverse. The variability in the ChatGPT Political texts corpus appears to be the largest, as indicated by its wider interquartile range and whisker length.

**Fig 2:**
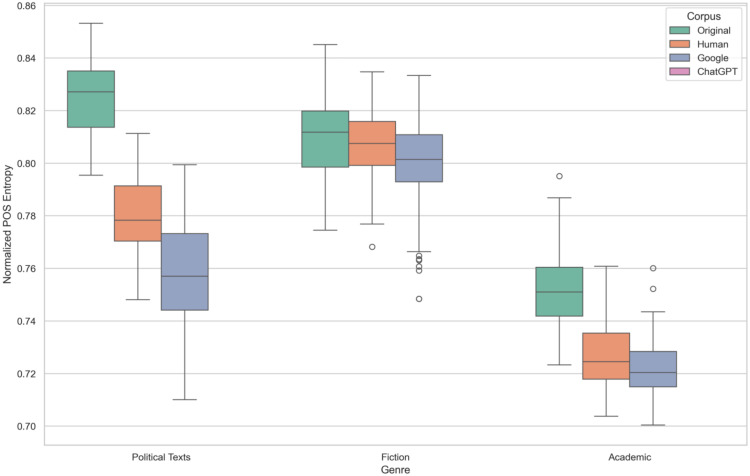
POS Entropy Across Corpus and Genres.

Fig 2 provides a compelling visualization of syntactic complexity across the different groups. The most striking pattern is the clear separation between the Original corpus and the three translated corpora (Human, Google, ChatGPT). The Original texts maintain a significantly higher median POS entropy in all genres, visually demonstrating that their syntactic structures are more complex and varied than any of the translated versions.

Among the translated corpora, the trend of syntactic simplification is evident. Human translations tend to have a slightly higher median POS entropy than Google and ChatGPT translations, especially in the Political texts and Fiction genres. Google and ChatGPT appear to produce texts with the lowest syntactic complexity, with their distributions often overlapping at the lower end of the scale.

Furthermore, a consistent hierarchical pattern emerges among the genres regardless of the corpus: Fiction texts consistently show the highest syntactic complexity, followed by Political texts, with Academic demonstrating the lowest complexity. The non-overlapping boxes between the three genres within the Original corpus, for instance, visually represent the statistically significant differences identified by the Kruskal-Wallis and post-hoc tests. This robust pattern suggests that genre conventions for sentence structure are a dominant factor influencing syntactic complexity.

For all significant results, a Dunn’s post-hoc test with Bonferroni correction was used for pairwise comparisons, with detailed results shown in Table 3. The analysis revealed a statistically significant effect of the translation modes on Normalized Unigram Entropy, H(3) = 122.99, p < .001 (see Table 4). Post-hoc comparisons showed that the Google corpus had significantly lower lexical diversity than the Original (Adjusted p < .001), Human (Adjusted p < .001), and ChatGPT (Adjusted p < .001) corpora. The Human corpus also showed significantly lower diversity than the Original (Adjusted p < .001) and ChatGPT (Adjusted p < .001) corpora. However, the difference between the Original and ChatGPT corpora was not statistically significant (Adjusted p > .99).

**Table 3:** Pairwise Comparisons from Dunn’s Post-Hoc Tests with Bonferroni Correction.

Dependent Variable	Comparison	Z-value	Adjusted p-value	Significant (p < .05)
Unigram Entropy by Corpus	Google vs. Original	−8.11	<.001	Yes
Unigram Entropy by Corpus	Google vs. Human	−4.43	<.001	Yes
Unigram Entropy by Corpus	Google vs. ChatGPT	−9.84	<.001	Yes
Unigram Entropy by Corpus	Human vs. Original	−5.47	<.001	Yes
Unigram Entropy by Corpus	Human vs. ChatGPT	−6.5	<.001	Yes
Unigram Entropy by Corpus	Original vs. ChatGPT	0.2	>.99	No
POS Entropy by Corpus	Google vs. Original	−9.92	<.001	Yes
POS Entropy by Corpus	Google vs. Human	−3.53	0.008	Yes
POS Entropy by Corpus	Google vs. ChatGPT	−2.16	0.483	No
POS Entropy by Corpus	Human vs. Original	−7.21	<.001	Yes
POS Entropy by Corpus	Human vs. ChatGPT	1.15	>.99	No
POS Entropy by Corpus	Original vs. ChatGPT	7.82	<.001	Yes
Unigram Entropy by Genre	Academic vs. Political Texts	−6.36	<.001	Yes
Unigram Entropy by Genre	Academic vs. Fiction	−1.54	0.324	No
Unigram Entropy by Genre	Political Texts vs. Fiction	4.38	<.001	Yes
POS Entropy by Genre	Academic vs. Political Texts	−6.95	<.001	Yes
POS Entropy by Genre	Academic vs. Fiction	−10.58	<.001	Yes
POS Entropy by Genre	Political Texts vs. Fiction	−4.61	<.001	Yes

Note: The Z-value represents the test statistic for each pairwise comparison. The p-value has been adjusted for multiple comparisons using the Bonferroni method.

**Table 4:** Results of Kruskal-Wallis H Test for Entropy Measures by Corpus and Genre.

Dependent Variable	Grouping Variable	H-statistic	df	p-value	Significant Pairwise Differences (Post-hoc)
Normalized Unigram Entropy	Corpus	122.99	3	<.001	(Original = ChatGPT)> Human > Google
Normalized POS Entropy	Corpus	133.56	3	<.001	Original > Human > Google; Human ≈ ChatGPT; Google ≈ ChatGPT
Normalized Unigram Entropy	Genre	40.52	2	<.001	Political texts> (Fiction = Academic)
Normalized POS Entropy	Genre	111.96	2	<.001	Fiction > Political texts> Academic

Note: Pairwise comparisons were conducted using Dunn’s post-hoc test. ‘ > ‘indicates a statistically significant difference (p < .05) where the group on the left has a higher median rank. ‘ = ‘indicates no significant difference between the groups.

Similarly, a significant effect of the translation modes was found on Normalized POS Entropy, H(3) = 133.56, p < .001. The post-hoc analysis confirmed that the Original corpus had significantly higher syntactic complexity than the Human (Adjusted p < .001), Google (Adjusted p < .001), and ChatGPT (Adjusted p < .001) corpora. Furthermore, Human translations demonstrated significantly higher syntactic complexity than Google translations (Adjusted p = .008). No significant differences were found between Human and ChatGPT translations (Adjusted p > .99) or between Google and ChatGPT translations (Adjusted p = .483).

Regarding genre, a statistically significant difference was found for Normalized Unigram Entropy, H(2) = 40.52, p < .001. Post-hoc tests revealed that Political texts had significantly higher lexical diversity than both Fiction (Adjusted p < .001) and Academic (Adjusted p < .001). The difference in lexical diversity between Fiction and Academic texts was not statistically significant (Adjusted p = .324).

Finally, the effect of genre on Normalized POS Entropy was also highly significant, H(2) = 111.96, p < .001. Dunn’s post-hoc tests indicated that all three genres were significantly different from one another: Fiction texts had significantly higher syntactic complexity than Political texts (Adjusted p < .001) and Academic (Adjusted p < .001), and Political texts had significantly higher complexity than Academic (Adjusted p < .001).

## 5. Discussion

The primary finding is that translated texts, regardless of modes (HT, NMT, ChatGPT), generally exhibit simplification compared to original non-translated texts, although the degree and nature of this simplification vary. A clear hierarchy of linguistic complexity emerged: original texts consistently displayed the highest complexity (highest lexical and syntactic entropy). Human Translations were generally simpler than originals. Both NMT systems and ChatGPT produced outputs simpler than both originals and HT, with NMT tending towards the greatest simplification, particularly at the lexical level as measured by unigram entropy. ChatGPT occupied an interesting position, often lexically more complex than NMT but less so than HT, while showing syntactic simplification (low POS entropy). Crucially, genre was found to significantly moderate these patterns, exerting a particularly strong influence on syntactic complexity (POS entropy), while translation modes had a more dominant effect on lexical complexity (unigram entropy).

A particularly noteworthy finding that challenges a monolithic view of the simplification hypothesis is the minimal degree of lexical simplification observed in the fiction genre. While Human Translations in fiction still showed slightly lower unigram entropy than the original English novels. This observed resistance to simplification in a creative genre is theoretically significant and aligns with research suggesting that translation universals are highly contingent on genre [16,29]. Several factors might explain this phenomenon. First, literary translators may consciously strive to maintain or even enhance lexical richness as a compensatory strategy to make up for inevitable losses in stylistic nuance or cultural specificity from the source text. Second, the source language itself may play a role. Chinese, a character-based language, often uses concise, meaning-dense characters and idioms. Translating these into English may necessitate the use of a wider and more varied vocabulary to fully unpack their semantic content, a process that counteracts simplification pressures. This aligns with findings by Williams [38], who also observed complexification in certain translation directions. Finally, the inherent creativity and stylistic freedom afforded by the fiction genre might empower translators to act more as creative co-authors than mere conduits, leading them to explore a broader lexical range than is typical in more constrained genres like academic or political texts. This specific result thus underscores that simplification is not an inevitable or uniform outcome of the translation process. Instead, its force appears to be mediated by genre, with creative contexts providing fertile ground for translators to preserve, and sometimes even elaborate on, lexical complexity.

The simplification observed in Human Translation aligns with well-established hypotheses in CBTS. This tendency can be attributed to cognitive factors inherent in the translation process, such as efforts to manage cognitive load, reduce ambiguity, enhance clarity for the target audience, and adhere to target language norms [5,10,49]. Translators might subconsciously opt for more frequent or structurally less demanding options, reflecting Pym’s [50] notion of risk aversion. The fact that HT in our study was less simplified than MT contradicts some previous findings using different metrics [24], underscoring the importance of how simplification is operationalized and measured. The more pronounced simplification found in NMT and ChatGPT likely originates from their fundamentally different, non-cognitive operating mechanisms. These systems optimize translations based on statistical patterns learned from vast datasets, often prioritizing fluency and probability over preserving source text complexity or nuanced lexical choices [25]. Algorithmic bias towards frequent patterns and the nature of training data, often skewed towards standardized language, can lead to outputs with reduced lexical and syntactic variation [23,43]. The specific profile of ChatGPT—slightly more lexically diverse than NMT but often syntactically simpler—might reflect its LLM architecture, enabling better contextual understanding and lexical access but potentially defaulting to more standardized syntactic structures during generation to ensure coherence across a wider range of inputs. This differs from the findings of Liu et al. [36] for HT (Chinese), where lexical simplification co-occurred with syntactic complexification (higher POS entropy), suggesting translation modes deeply influences simplification patterns across linguistic levels.

Our results strongly corroborate the significance of genre as a variable modulating translation features, a point emphasized by Kruger and van Rooy [29] and Liu and Afzaal [16]. The finding that genre significantly affects syntactic complexity (POS entropy) more than lexical complexity (unigram entropy) across all text varieties is particularly noteworthy. Academic genre, characterized by conventional structures, consistently showed lower syntactic entropy, whereas political texts, perhaps demanding more structural adaptation for clarity, exhibited higher original complexity but underwent significant syntactic simplification in translation across all modes. This suggests that genre-specific communicative norms and structural constraints can override or interact strongly with mode-specific simplification tendencies. This aligns with House’s [51] caution that some perceived translation universals might reflect broader patterns conditioned by register or genre rather than the translation process per se. The consistent simplification in academic genre across modes, for instance, might be driven more by the genre’s inherent structural constraints than by translation itself.

These findings contribute significantly to the ongoing debate surrounding translation universals (TUs), particularly the notion of simplification. While corroborating a general tendency toward simplification in translated as opposed to original texts [5,17], our results challenge the idea of simplification as a monolithic or truly universal phenomenon. Instead, the systematic variation observed across translation modalities and genres underscores the contingent and probabilistic nature of TUs [52,53], with our study providing quantitative evidence for the conditioning roles of mode and genre. These findings contribute significantly to the debate on translation universals (TUs) by moving beyond a simple confirmation or rejection of simplification. Instead, our results call for a more granular, modal theory of translation. This perspective posits that the very generative mechanism of each translation mode fundamentally shapes its linguistic output. For instance, Human Translation (HT) is driven by human cognition, involving risk aversion [50], a desire for clarity, and creative problem-solving. This often leads to a moderate simplification, balancing fidelity with target-language norms. Neural Machine Translation (NMT), in contrast, operates on statistical optimization, learning frequent patterns from its training data. This can result in a more pronounced simplification, particularly lexically, as the system defaults to high-probability, common vocabulary and structures to ensure fluency [25,43].

LLM-based translation, exemplified by ChatGPT, introduces a third paradigm. Its generative process is based on vast contextual inference, allowing it to access a wider lexical repertoire, thus resisting lexical simplification as our data shows. However, to maintain coherence across long-range dependencies, it may default to more standardized syntactic structures, leading to syntactic simplification. Therefore, the distinct entropy profiles we observed are not random variations but are the predictable linguistic ‘fingerprints’ of their underlying modes. This modal approach challenges the notion of a singular “third code” [7], suggesting instead a spectrum of ‘translationese,’ each defined by its technological or cognitive origin.

Furthermore, the differential impact of genre on lexical versus syntactic entropy indicates that TUs such as simplification may not operate uniformly across linguistic levels [36], warranting further exploration of potential dissociations among lexical, syntactic, and discursive features across modes and genres. Finally, this study validates the entropy-based approach [36] as a refined and effective lens for corpus-based translation studies (CBTS), offering a global, information-theoretic measure sensitive to distributional patterns, and thereby overcoming limitations of traditional metrics [21,38] while reconciling contradictory findings from studies relying on isolated indicators.

## 6. Conclusion

This study revealed three major patterns in translation simplification across modes and genres. First, a clear hierarchy of linguistic complexity emerged across translation modes, with original texts showing the highest complexity, followed by Human Translations, ChatGPT translations, and Google translations. This pattern held true for both lexical and syntactic complexity measures, though with varying degrees of significance. Second, genre effects manifested differently at lexical and syntactic levels. While genre had limited impact on lexical complexity, it strongly influenced syntactic complexity. Academic genre consistently demonstrated lower syntactic complexity across translation modes, while novel texts maintained higher complexity levels, particularly in machine translations. Third, translation modes exhibited distinct genre-specific patterns. Political texts showed the most pronounced differences between original and translated texts, particularly in machine translations. Academic genre demonstrated the most consistent simplification pattern across all modes. Most notably, ChatGPT translations maintained more consistent levels of complexity across genres than other translation modes, suggesting a distinct translation strategy.

Our findings significantly advance the theoretical understanding of translation universals in three key ways. First, the study demonstrates that simplification, rather than being a uniform universal feature, manifests as a complex, probabilistic phenomenon that varies systematically across translation modes and genres. This supports the shift from Baker’s [5] original conceptualization of translation universals toward what Toury [53] and Chesterman [52] describe as probabilistic laws, while suggesting an even more nuanced reality where these probabilities are modulated by both mode and genre. Second, the emergence of ChatGPT as a distinct translation mode with its own complexity patterns challenges traditional binary distinctions between translated and non-translated texts. The observed hierarchy of linguistic complexity suggests the need for what might be termed a “modal theory of translation” that can account for the distinctive features of different translation processes. This extends beyond Frawley’s [7] concept of the “third code” to suggest a spectrum of translational varieties, each with its own complexity signature. Third, the differential effects of genre on POS entropy versus unigram entropy reveal that simplification operates differently at lexical and syntactic levels, challenging assumptions about the uniformity of translation universals. This finding supports House’s [51] argument that some observed translation behaviors may reflect broader patterns of language use rather than translation-specific phenomena.

The study validates the effectiveness of entropy-based measures for analyzing translation complexity, addressing the limitations of traditional metrics noted by researchers from Williams [38] to Liu and Afzaal [16]. The ability to capture both lexical and structural complexity simultaneously represents a significant methodological advance, offering a more robust framework for future translation studies research. This approach has proven particularly valuable in revealing the subtle interactions between translation mode and genre that might be missed by conventional metrics.

For translation practice and technology development, our findings have several important implications. The discovery that ChatGPT translations maintain more consistent levels of complexity across genres while showing distinctive patterns from both human and traditional machine translations suggests the emergence of a new paradigm in translation technology. This has implications for how we approach translation quality assessment and the development of future translation systems. The strong genre effects observed, particularly in technical texts, suggest the need for genre-sensitive approaches to both human and machine translation. This aligns with what Kruger and van Rooy [29] identified as the crucial role of genre in translation features, while extending their insights to include AI-mediated translation.

Future research might explore how these patterns evolve as AI technology advances, how they manifest across different language pairs, and how they relate to other translation universals such as explicitation and normalization. As Zhang and Toral [54] note, the rapid evolution of AI systems presents both opportunities and challenges for translation studies, suggesting the need for longitudinal studies tracking changes in translation patterns over time.

The field may be approaching what could be termed a “modal turn” in translation studies – a recognition that different translation modes (human, traditional machine, and AI-mediated) represent fundamentally different approaches to the translation task, each with its own patterns of linguistic behavior. This suggests the need for what De Sutter et al. [48] call a multi-methodological, multifactorial approach to translation research, one that can capture the increasing complexity of contemporary translation practices while maintaining theoretical coherence.
